# Searching for Protein Off-Targets of Prostate-Specific Membrane Antigen-Targeting Radioligands in the Salivary Glands

**DOI:** 10.1089/cbr.2024.0066

**Published:** 2024-12-04

**Authors:** William Julian, Olga Sergeeva, Wei Cao, Chunying Wu, Bernadette Erokwu, Chris Flask, Lifang Zhang, Xinning Wang, James Basilion, Sichun Yang, Zhenghong Lee

**Affiliations:** ^1^ Radiology Department, Case Western Reserve University, Cleveland, Ohio, USA.; ^2^ Biomedical Engineering Department, Case Western Reserve University, Cleveland, Ohio, USA.; ^3^ Nutrition Department, Case Western Reserve University, Cleveland, Ohio, USA.

**Keywords:** PSMA, targeted radioligand therapy, off-targets, QSAR

## Abstract

**Background:**

Prostate specific membrane antigen (PSMA)-targeted radioligand therapies represent a highly effective treatment for metastatic prostate cancer. However, high and sustain uptake of PSMA-ligands in the salivary glands led to dose limiting dry mouth (xerostomia), especially with α-emitters. The expression of PSMA and histologic analysis couldn’t directly explain the toxicity, suggesting a potential off-target mediator for uptake. In this study, we searched for possible off-target non-PSMA protein(s) in the salivary glands.

**Methods:**

A machine-learning based quantitative structure activity relationship (QSAR) model was built for seeking the possible off-target(s). The resulting target candidates from the model prediction were subjected to further analysis for salivary protein expression and structural homology at key regions required for PSMA-ligand binding. Furthermore, cellular binding assays were performed utilizing multiple cell lines with high expression of the candidate proteins and low expression of PSMA. Finally, PSMA knockout (PSMA−/−) mice were scanned by small animal PET/MR using [^68^Ga]Ga-PSMA-11 for in-vivo validation.

**Results:**

The screening of the trained QSAR model did not yield a solid off-target protein, which was corroborated in part by cellular binding assays. Imaging using PSMA−/− mice further demonstrated markedly reduced PSMA-radioligand uptake in the salivary glands.

**Conclusion:**

Uptake of the PSMA-targeted radioligands in the salivary glands remains primarily PSMA-mediated. Further investigations are needed to illustrate a seemingly different process of uptake and retention in the salivary glands than that in prostate cancer.

## Introduction

Recent clinical studies using molecularly targeted radioligands have shown remarkable efficacy in the treatment of metastatic prostate cancer.^1^ The therapeutic radioligands comprising a short peptide targeting prostate-specific membrane antigen (PSMA) and when chelated with α-emitting actinium-225 (^225^Ac) have demonstrated remarkable clinical results in patient end-stage prostate cancer, including achieving complete imaging and biochemical responses following microdosing.^2,3^ Despite the encouraging results, patients reported having dry mouth (xerostomia), leading in severe cases to discontinuation of the treatment.^4^ Positron emission tomography (PET) scans performed using similar PSMA-targeting radioligands chelated with Gallium-68 (^68^Ga) demonstrated strong and persistent uptake in all salivary glands.^5^ However, RNA-sequencing of salivary tissues demonstrated weak expression of PSMA, which could not directly explain the toxicity.^6–8^ Furthermore, patchy histological staining of PSMA in salivary tissue has led to an alternative hypothesis that the radioligand uptake might be non-PSMA mediated, suggesting that PSMA-ligands are binding to off-target protein(s) in the salivary glands, leading to the dose-limiting toxicity.^4,8,9^ In this report, the authors summarize their efforts in seeking these possible off-target(s).

*In silico* techniques, including structure-based virtual screening and computer-aided drug design involve computation of the interaction of a target protein with screening ligands and represent alternatives to traditional wet-lab methods for new drug discovery or repurposing existing drugs.^10–12^ Reverse virtual screening, a process in which the ligands are known, but the protein targets mediating their drug effects are not, represents the inverse of the technique.^13^ Both virtual screening and reverse virtual screening utilize the principles relating binding affinity and free energy of binding to screen for molecules or targets from a constructed database of computed binding affinities linked to physicochemical descriptors of the protein–ligand pairs.^14^ The framework of their reverse virtual screening for potential off-target(s) of the known PSMA-ligands is based on reversed use of the quantitative structural activity relationship model (QSAR), which depicts the relationship between target (protein) and ligand.^15^ In their QSAR model, they relate a series of physicochemical feature descriptors describing protein–ligand pairings (***s***tructure) to a set of calculated binding affinities for the protein–ligand pairs (***a***ctivity or property), allowing for the trained QSAR model to algorithmically predict the interaction between protein and ligand without requiring computationally intensive free energy-binding simulations for all protein–ligand pairs.^16^ While most QSARs process a list of potential ligands for a known target, they construct the QSAR to appraise unknown protein off-target(s) against a set of bait PSMA-ligands, representing a type of reverse virtual screening.

Our QSAR model specific to the PSMA-radioligands uses protein sequence-derived and ligand structure-based feature descriptors to screen protein repositories representing likely protein off-targets for the known PSMA-radioligands. The specificity of PSMA-targeting radioligands is due to strong intermolecular forces between the key amino acid residues in the PSMA-binding pocket and the radioligand’s targeting motif,^2,17,18^ with ligand activity in the nanomolar concentrations, corresponding to significant negative free energies of binding.^19^ Due to the scanty experimental results on PSMA-radioligand binding to non-PSMA proteins, molecular dynamics simulations can establish a set of protein–ligand binding energies, which serve as a proxy for biologically determined protein–ligand binding affinity for machine learning. These calculated binding energies and the corresponding physicochemical features represent the “***A***ctivity” and “***S***tructure” components for the QSAR model.^20^ Once learned, the QSAR model can be used to screen a large number of proteins based only on protein sequences to establish a pool of potential targets. This pool of potential targets could then be refined by comparison with proteins that are selective or highly expressed in the salivary gland using databases of mRNA and protein expression for salivary gland cells to further select for protein targets. In addition, pairwise protein alignments could then be used to look for overlap at key amino acid residues, particularly those involved in substrate stabilization.

To validate the *in-silico* results, they utilized cellular and animal models to measure the binding and uptake of radioligands in representative models. A variety of cellular lines with known protein expression were tested for *in vitro* radioligand uptake. PSMA-knockout mice were also used for *in vivo* evaluation of radiotracer uptake, both of which complemented *in-silico* investigations.

## Methods

### Target ligands

The three target ligands included 2-PMPA, MUD, and DCFPyL. The 2-PMPA, also known as 2-(phosphonomethyl)-pentanedioic acid, is phosphonate-based derivative of glutamate originally designed as an GCP II inhibitor.^21^ MUD, known as (S)−2-(3-((R)−1-carboxy-2-methylthio)ethyl)ureido)-pentanedioic acid or DCMC, is a urea-based GCPII inhibitor.^22^ DCFPyL, also known as 2-(3-(1-carboxy-5-[(6-[^18^F]fluoro-pyridine-3-carbonyl)-amino]-pentyl)-ureido)-pentanedioic acid, is a second-generation urea-based GCPII ligand.^23^ Ligand structures were obtained from the RCSB and ChemBL databases Supplementary Data S1.^24,25^

### Unknown targets

Protein structures for all target proteins were obtained from the RCSB database.

### Molecular docking

Molecular docking was carried out to position the ligands for molecular dynamics simulations. Three ligands, including 2-PMPA, MUD, and DCFPyL, were selected for molecular docking with target proteins. Chemical structures of ligands were retrieved from RCSB and ChEMBL databases and prepared using CCDC Mercury software.^25,26^

Docking was performed using the GOLD software package with default parameters unless otherwise specified.^27^ The GOLD algorithm employs a genetic algorithm to explore the conformational space of ligands within the predefined receptor grid. The top-ranked pose represented by scoring function were chosen for each of the three ligands for usage in molecular dynamics simulations.

The ChemPLP scoring function was employed to evaluate the binding affinity of ligands within the binding site.^28^ The ChemPLP is a combination of piecewise linear potential combined with torsional, hydrogen bonding, and metal coefficients. The top-ranked ligand poses were visually inspected to ensure proper orientation within the binding site. Swiss PDB Viewer was used for the addition of missing protein residues after docking was completed.

Target protein structures for *post hoc* docking were obtained from the RCSB protein database and prepared by removing water molecules and adding hydrogen atoms using GOLD or Schrödinger-2023–3.^29^ The active site, including the pocket comprising E272, N379, R389, R387, and R319, was defined based on experimental binding-site information and/or the positioning of external ligands within the protein structure and verified through literature review.

### Molecular dynamics (MD) simulations

Three-dimensional (3D) structure of the protein–ligand complex was obtained from molecular docking as described above. Protein–ligand complexes were simulated using 4 repeats with both Charmm36 and Amber forcefields. The ligand topology and parameter files were generated using the acpype parameterization tool for Amber forcefields, and CgenFF tool was used for parameterization of the ligand for Charmm forcefields.^30,31^

The protein–ligand complex was solvated in a dodecahedral periodic box with TIP3P water molecules with a box size proportional to the max diameter of the complex. Appropriate counterions were added to maintain system neutrality to a concentration of 0.15M. The system was energy minimized to remove steric clashes and achieve a stable starting structure. The prepared system was subjected to energy minimization using the steepest descent algorithm until convergence was achieved, ensuring a force tolerance below 1000 kJ mol^−1^ nm^−1^.

The system was equilibrated in the canonical (NVT) and isothermal–isobaric (NPT) methods to stabilize temperature and pressure, respectively. The Berendsen thermostat and Parrinello Rahman barostat were employed during these equilibration steps. An MD simulation was performed using the leap-frog integration scheme with a time step of 2 fs. Simulations were performed for 10 ns.

The CHARMM36 and Amber force fields were utilized for the protein, and ligand parameters were retained from the setup phase. Nonbonded interactions were treated with a cutoff of 1.2 nm, and long-range electrostatics were computed using the Particle Mesh Ewald (PME) method. The system was maintained at a constant temperature of 300 K using the modified Berendsen thermostat and a pressure of 1 bar using the Parrinello–Rahman barostat. Coordinates, velocities, and energies were saved at every 50 ps during the production run for subsequent analysis.

The free energy of binding (ΔG_bind) was calculated using the GMX_MMPBSA tool.^32^ Free energy of binding was calculated as the sum of the molecular mechanics energy (ΔE_MM), solvation free energy (ΔG_solv), and entropy contribution (−TΔS) for the given protein–ligand complex. All calculations were performed using generalized born parameters.

All MD calculations and free energy calculations were performed on the High-Performance Computing Resource in the Core Facility for Advanced Research Computing at Case Western Reserve University. Docking simulations were performed on Intel i7-10700 CPU processor.

### QSAR

Data of ***A***ctivity (or property) for QS***A***R modeling was obtained from the output of free energy of binding calculations following MD simulations. These data included a set of selected proteins, three ligands, and their associated experimental free energies of binding.

Data of ***S***tructure for Q***S***AR modeling came from the protein–ligand pairs, for each of which, a set of descriptor values was calculated for both the protein and the ligand. Ligand descriptors were calculated using the rCDK package using the R software, resulting in each of the three ligands having 197 descriptors that consisted of physicochemical properties, graph theoretical indices, and functional group counts.^33^ Protein descriptors were calculated using the iFeature program, resulting in each protein being characterized with 13,494 descriptors, including amino acid composition, dipeptide composition, autocorrelation descriptors, quasi-sequence order, amphiphilic pseudo amino acid composition, and total amino acid properties.^34^ (The two long lists of descriptors enumerating ligand and protein structural features, respectively, are available upon request.) Proteins under 30 AA in length were excluded from analysis given the constraints of protein characterization requiring sequence length >30 AA.

Each resulting protein–ligand pair comprising the concatenated protein–ligand descriptors underwent principal component analysis (PCA) to identify a much smaller subset of descriptors. The resulting dataset with selected PCA components was randomly split into training (75% of the data) and testing sets (25% of the data) to evaluate model performance.

QSAR models were built using the tidymodels framework in R. The model formula was defined with the dependent variable (binding energy) and independent variables (molecular descriptors). Different models, such as linear regression, decision trees, or random forests, were considered based on the nature of the data. Data preprocessing steps, such as centering and scaling of numerical predictors, were applied to ensure numerical stability and comparability.

The model’s performance was evaluated on the testing set using R-squared and root mean Square error (RMSE). The analysis was conducted using R version 4.3.2.

### Library screening

Protein libraries from the human protein atlas and Uniprot databases comprising secreted and membrane proteins were downloaded and screened using the model following characterization using the methods described previously.^35,36^ The 2-PMPA was used as the screening ligand to calculate binding energies. Proteins with binding energies in the top 25% of screened proteins were designated target candidates and subject to cross-validation with databases of salivary protein expression. High or selective expression was defined by having 4-fold higher expression in the salivary gland compared with other tissues, expression limited to the salivary gland or expression in the salivary gland and less than 1/3 of human tissues. Proteins identified by the model as targets and meeting expression criteria were selected for further analysis through pairwise protein alignments.

### Pairwise protein alignments

Following protein screening, proteins designated as potential targets were compared with databases of proteins known to be selectively or highly expressed in the salivary gland relative to other tissues. Proteins designated as targets found to be highly or selectively expressed in the salivary gland were subjected to pairwise alignment using the EMBL-EBI pairwise sequence alignment tool.^37^ Sequences were compared against the FOLH1 protein sequence at 18 separate amino acids comprising four major structural features of the PSMA binding pocket. Sequences with >60% sequences homology at any of the 4 major structural features were simulated using MD simulations as described earlier.

### Cellular/animal models

*In vitro* studies were carried out to test PSMA-ligand binding to possible non-PSMA protein targets used in the training set. Cell lines, from ATCC, with low PSMA expression were selected for ligand-binding assays, and possible non-PSMA targets with high expression in each cell line are listed in Table 1. The RT16 and D4 cell lines, which were engineered from the parent R2 (CHO) cell lines to express human folate receptors α and β, respectively, were generously given by Dr. Larry H. Matherly from Karmanos Cancer Institute at Wayne State University.^38^

**Table 1.** Cell Lines Expressing Candidate Protein Targets

Cell line	Target(s)	Notes
RT4	GRIP1	Glutamate receptor interacting protein 1
Capan2	SLC7A11	Cystine/glutamate transporter
KB	SLC1A1 & FOLR1	Excitatory amino acid transporter 3 (EAAT3) and folate receptor alpha
HEK293	CA2 & AQP6	Carbonic anhydrase 2 and aquaporin 6
HepG2	FTCD	Formimidoyltransferase cyclodeaminase
HeLa	AQP3 & FOLR1	Aquaporin 3 and folate receptor alpha
WM-115	GRIK2	glutamate receptor, ionotropic, kainate 2, EAA4, or GluR6
U-266	SLC14A2	Urea transporter 2 (UT2 or UT-A)
Caco2	SLC46A1	Proton-coupled folate transporter (PCFT)
RPTEC	TMEM213	Transmembrane protein 213 (highly enriched in the salivary glands)
R2	PCFT−/−, FOLR−/−	Chinese Hamster ovary cells (CHO), the parent cell line of D4 and RT16
RT16	FLOR1	R2 engineered to express human folate receptor alpha
D4	FOLR2	R2 engineered to express human folate receptor beta

For each cell line, cells were incubated with different amounts of H-3 labeled simplest PSMA radioligand (S)−2-[3-{(S)−5-amino-1-carboxypentyl}ureido]-pentanedioic acid ([3H]ZJ-24, RC TRITEC AG, Teufen, Switzerland) in the range of tracer dose for 60 min followed by washing, palleting, and liquid scintillation counting to measure total uptake; the other portions had 10 μM cold PSMA-ligand ZJ-24 added for measuring nonspecific uptake through competition. A subtraction yielded specific uptake.

*In vivo* small animal PET/MR imaging using the clinical ligand [^68^Ga]Ga-PSMA-11 was performed with 3 PSMA null (PSMA−/−) mice, in which PSMA expression is disabled and compared with 3 wild-type (wt) mice for radioligand uptake in the salivary glands.^39^ Mouse scans were performed on 9.4T Bruker Biospec preclinical MRI scanner (Bruker Corp., Billerica, MA, USA). Each mouse was anesthetized with isoflurane and positioned within a Cubresa NuPET PET insert and PET-MRI compatible radiofrequency volume MRI coil (ID = 35 mm). Around 200 μCi (7.4 MBq) of the radioligand [^68^Ga]Ga-PSMA-11 was injected intravenously through the tail vein. Five-minute static small animal PET scans were acquired with the NuPET insert at 0.5 and 1.0 hour postinjection (some animals only had 1-postinjection scans). Following initial localizer scans, mouse MR images were acquired simultaneously with a coronal 3D True FISP (Fast Imaging with Steady-State Free Precession) acquisition (FOV = 60 × 30 × 30 mm, matrix size = 256 × 128 × 128, TR/TE = 4.0/2.0 ms, flip angle = 30 degrees, 5 signal averages, scan time = 6 min). After scanning, MR and PET scans were aligned for region definition and quantification, i.e., calculation of region-based standardized uptake values (SUVs) such as SUV_max_ and SUV_peak_.^40^ All animal procedures were approved by the university Institutional Animal Care and Use Committee (IACUC) under the IACUC protocol 2015–0123.

## Results

### Construction of learning set

Seventy-nine candidate protein targets were obtained from the RCSB database. Preference was given to structures with intrinsic ligands to maximize positioning of the PSMA-ligands. Each protein was docked with all 3 of the bait ligands. Following docking, MD simulations were performed as described earlier and the MMPBSA software was used for determination of the free energy of binding. Simulation-derived binding affinities were calculated using 238 total protein–ligand pairs. Binding energies ranged from −1.635 kJ/mol to a maximum of −46.085 kJ/mol (Supplementary Table S1). Median binding energy was −17.935 kJ/mol, with an IQR of 14.62.

Training and test datasets for the QSAR model were constructed using a 75%/25% split (training/test) with stratification to training and test sets based upon binding energy (response) using four bins. The training set included 177 protein–ligand pairs. Median free energy of binding was −17.935, with a min of −46.015 and a max of −1.635. The test set included 60 protein–ligand pairs with a median free energy of binding of −17.73 kJ/mol, ranging from −46.09 kJ/mol to −2.14 kJ/mol. The model was trained explicitly on the training dataset. The test dataset was used only for evaluation of the trained model.

### Model characteristics

Following initial testing, a random forest model using 34 principal components was selected among other preliminary models based on RSME and *R*^2^ metrics to be used for the production runs. The 34 principal components represented 70.8% of the cumulative variance of the original data.

The random forest model comprising 34 principal components was trained on a dataset comprising molecular descriptors and experimentally determined binding affinities. The predictive performance of the model was evaluated using various metrics, including the RMSE, and coefficient of determination (*R*^2^).

**FIG. 1.**
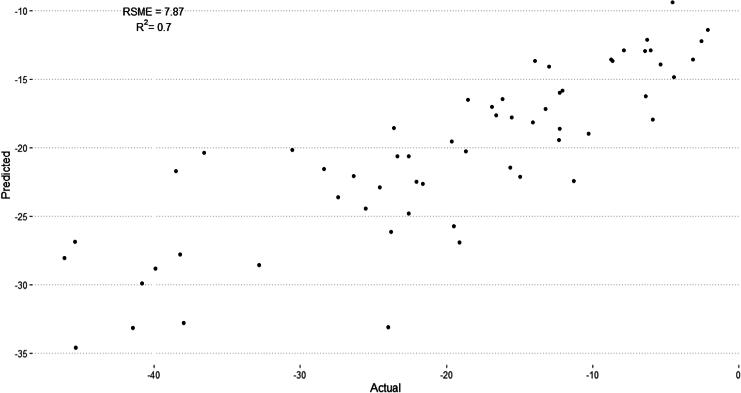
Comparison of predicted binding energies (Y-axis) from trained QSAR model and calculated data (X-axis) from molecular dynamics simulations. Sixty protein–ligand pairs in test dataset for QSAR model using 34 principal components and random forest architecture.

The RMSE of the model on the test dataset was found to be 7.75 kcal/mol, suggesting a reasonable accuracy in predicting the variability of binding affinities (Fig. 1). The *R*^2^ value of 0.70 indicated a medium correlation between the predicted and actual binding affinities, highlighting that the random forest model could capture the underlying patterns in the dataset. These characteristics of the model were deemed sufficient for screening databases for potential targets.

### Results of database screening

Plasma membrane and secreted proteins were selected from the uniport and human protein atlas databases using their respective keywords and/or identifiers. In total 9,128 proteins underwent characterization with feature descriptors and PCA in preparation for modeling as mentioned earlier. Median predicted free energy of binding for the modeled proteins was −15.109, with minimum values of −32.82 kJ/mol and maximum values of −5.87 kJ/mol. Proteins with predicted free energies of binding below the 25th percentile (−16.53 kJ/mol) were further characterized by comparison to gene and protein expression scores specific to the salivary gland (Supplementary Table S2). (Full data sets for all screened proteins are available upon request.)

### Pairwise comparison

**FIG. 2.**
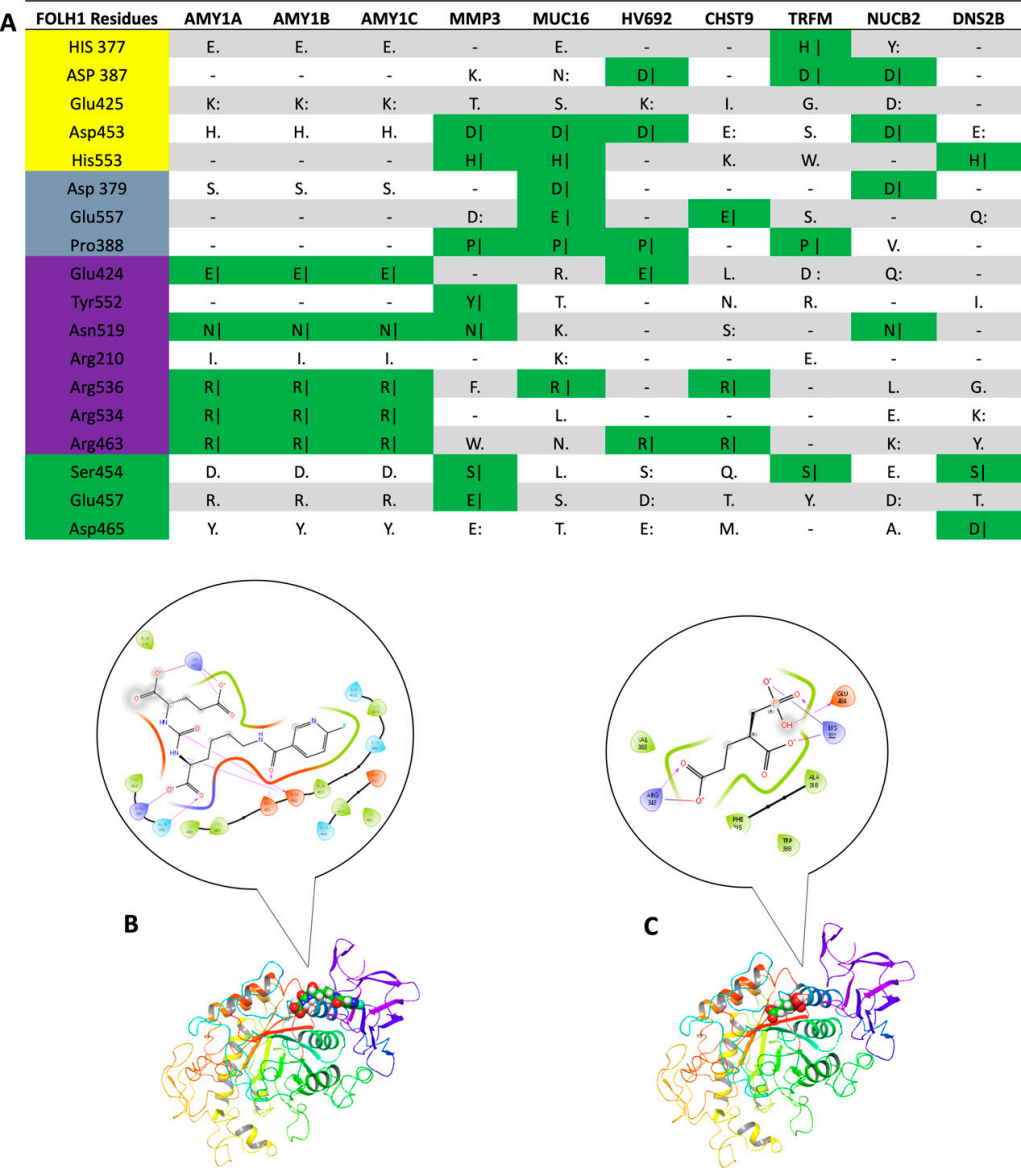
Pairwise protein alignments of target proteins proposed by model and FOLH1 by residue. **(A)** Top 10 proteins by homology at key amino acid residues following stratification by salivary gland RNA expression shown. Green indicates identical residues. FOLH1 protein residues colored based on proposed role: yellow, zinc binding; purple, substrate-binding residues; gray, residues with structural roles; green, residues that stabilize arginine patch.^46^ **(B, C)** Docking of DCFPyL and 2-PMPA into amylase 1 (PDB: 1C8Q), respectively. The upper portion of each indicates possible interactions between the PSMA-ligand (DCFPyL or 2-PMPA) and likely matching AA residues on amylase.

The resulting “potential targets” comprised 2,281 human proteins (the list is available upon request) and were compared with gene expression profiles of the salivary gland. Filtering of potential targets was performed by comparison against proteins known to be selectively or highly expressed within the salivary gland.^35,41^ Comparison of these target candidate proteins against those known to be highly or selectively expressed within the salivary gland yielded 81 matches (Supplementary Table S2), which were subjected to pairwise alignments. The top 10 aligned proteins were displayed in Figure 2A, from which only three proteins, AMYL1A, AMY1B, and AMY1C, showed significant sequence homology with the PSMA substrate-binding residues. However, further molecular simulations using the bait ligands docked to AMY1A structure produced free energy of binding of −7.26 kJ/mol for 2-PMPA, demonstrating poor overall binding while molecular docking revealed (Figs. 2B, 2C) rather shallow attachment between the PSMA-ligand (2-PMPA) and the imagined binding pocket on the amylase based on pairwise alignment, which is not the binding site for its native ligand.

As glutamate is part of all PSMA-ligands for binding and PSMA also possesses enzymatic activity toward folic acid, a subset of glutamate receptors and transporters as well as folate receptors and transporters were analyzed and summarized in Supplemental Supplementary Table S3. As there was no outstanding target from the list in terms of binding affinity for the PSMA-ligands, cell-binding assays were selectively performed next to confirm the negative binding from MD simulations.

### Cell-binding assays and animal scanning

**FIG. 3.**
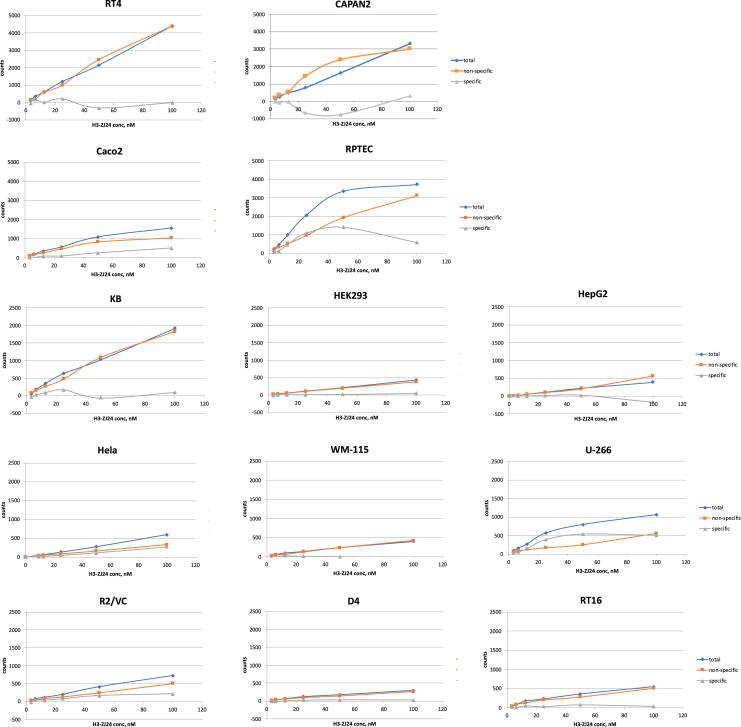
Cellular binding assays with PSMA-ligand [^3^Hs]ZJ-24 showed no specific uptake of the PSMA-targeting radioligand in any cell lines. Cell lines with high expression of the candidate target proteins and low expression of PSMA were used for binding assays.

Cells enriched with SLC1A1 (KB), SLC7A11 (Capan2), GRIP1 (RT4), FTCD (HepG2), AQP3 (HeLa), SLC14A2 (U-266), FOLR1 (RT16), and FOLR2 (D4), all showed no specific uptake of [3H]ZJ-24 (calculated as specific uptake = total uptake—nonspecific uptake) and shown in Figure 3.

**FIG. 4.**
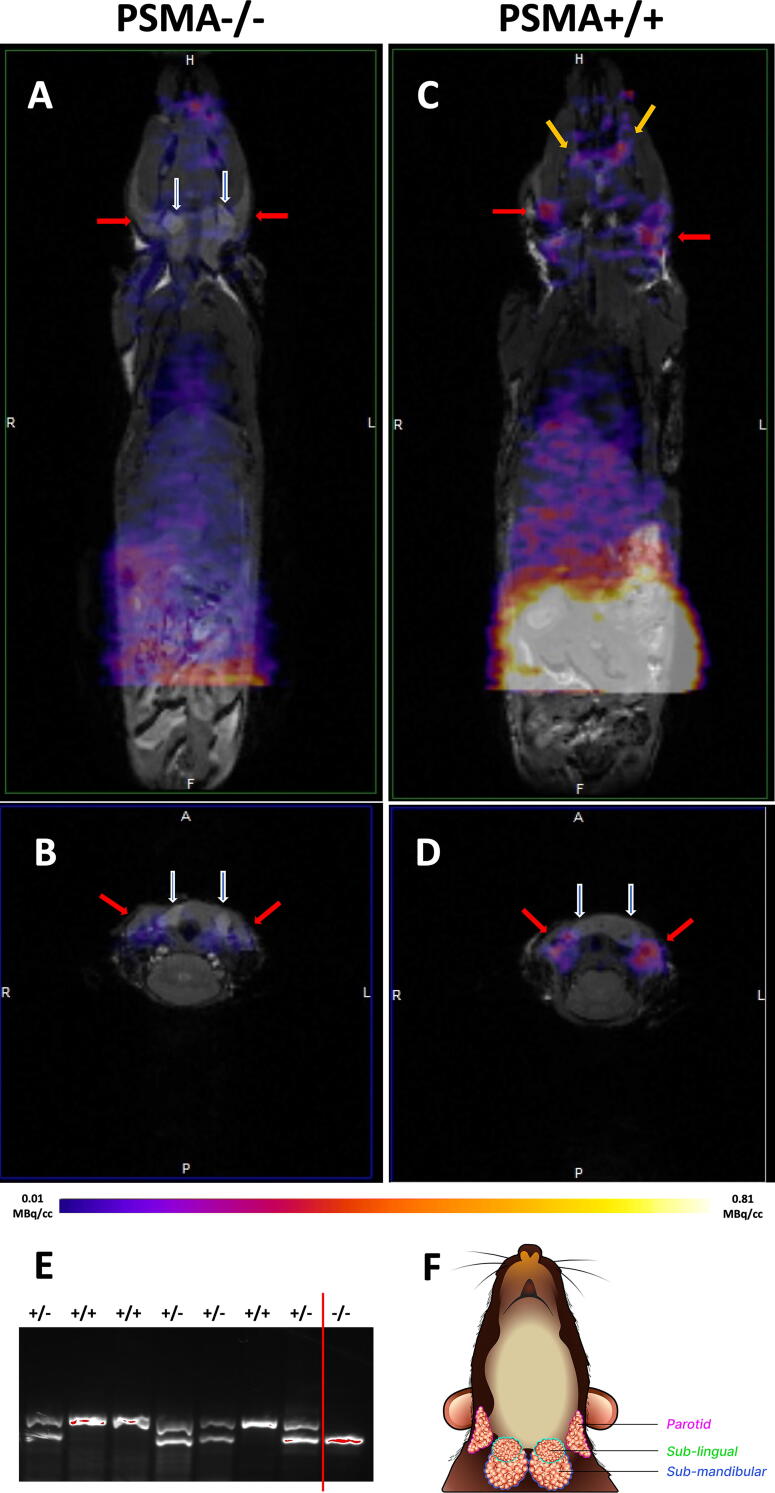
Comparison of [^68^Ga]Ga-PSMA-11 uptake between PSMA null (PSMA−/−) and wild-type (wt) mice. **(A, C)** coronal and **(B, D)** axial overlays of PET/MR images. The red arrows point to the parotid glands; the white arrows to sublingual glands in front of the submandibular glands; the orange arrows point to the lacrimal glands. **(E)** showed genotyping of several different mice to demonstrate knockout (PSMA−/−), heterozygosity (+/−), or wild-type (+/+) status; to right of red vertical line is the knockout (PSMA−/−). **(F)** Illustration of the major salivary glands in the mouse for orientation with the MR images.

Imaging using small animal PET with [^68^Ga]Ga-PSMA-11 showed overall low uptake in the knockout (PSMA−/−) mice in comparison with the wild-type (PSMA+/+) mice, as shown in Figure 4. Uptake in the parotid glands of PSMA null mice was about 37%–42% of that in the wild-type mice at 1.0 hour postinjection (SUVs of PSMA−/− vs. wild type), as shown in Table 2. Uptake in the sublingual and submandibular glands were not noticeable while uptake in the lacrimal glands was only noticeable in the wild-type mice, as shown in Figure 4. However, the vascular phase is not over by 1-h postinjection, and ligand retention (through internalization) cannot be assayed by Ga-68 PET imaging.

**Table 2.** Comparative Uptake of [^68^Ga]PSMA-11 in PSMA Null (PSMA−/−) and Wild-Type Mice

	Muscle background		Right parotid		Left parotid	
SUVpeak/std						
PSMA−/−	0.0539037	0.021831	0.0935844	0.0024531	0.0899706	0.007456
Wild-type	0.1513213	0.077567	0.22119358	0.0404992	0.2467296	0.0525369
SUVmax/std						
PSMA−/−	0.0592641	0.023289	0.10068921	0.0015846	0.0993156	0.0097529
Wild-type	0.1604765	0.076283	0.23704056	0.0411132	0.2627125	0.0512088

*Note*: *n* = 3 for each group were used to calculate the standard deviations (std). PSMA, prostate-specific membrane antigen.

## Discussion

PSMA-targeted radioligand therapies have shown remarkable efficacy in the treatment of advanced metastatic prostate cancer, yet their uptake and retention in the salivary glands have been the source of dose-limiting toxicity related to severe xerostomia.^7^ It has been speculated that this uptake could be mediated by a cellular non-PSMA off-target. But, their screening analysis using a unique PSMA-specific QSAR model validated in part by cellular-binding assays calls into question the existence of such a protein off-target. In addition, in-vivo experiments using PSMA knockout (PSMA−/−) mice demonstrated significantly less uptake in the salivary glands than in wild-type individuals. The lack of uptake within the submandibular and sublingual glands and reduced (37–42% of the wild-type) uptake in parotid glands of PSMA−/− mice suggest that the uptake in the salivary glands is still somehow primarily PSMA mediated, while nonspecific uptake may play a minor role.^42^ Establishing PSMA as the likely primary mediator of salivary gland uptake is important, given ongoing efforts to block this uptake, as well as the prognostic implications of salivary and tumor PSMA expression.^43^

While the negative results from this study support PSMA mediation of salivary gland uptake, the authors must also discuss the strengths and limitations of this study. Their approach was consistent with prior examples of reverse virtual screening, while specific to the PSMA-targeting radioligands.^13,44^ Given the paucity of knowledge regarding the interaction of PSMA ligands with the unknown non-PSMA proteins, the method was influenced by the knowledge of molecular interactions of PSMA-ligands with PSMA itself, and a similar model of binding for any off-target proteins was assumed, for which molecular docking, MD simulation, and screening of secreted and membrane proteins could recreate the potential steps of ligand binding to potential off-targets, even though the same ligand can bind to different proteins without structural similarity. It was also assumed that the potential off-target would be selectively or highly expressed in the salivary gland, as a widely expressed protein could not explain the selective uptake demonstrated in human studies.

MD simulation-calculated binding affinities were used to substitute the ground truth activity measurements during the training of the QSAR model, which introduces noise in the learning process. Although MD computation of the free-energy release during protein–ligand binding demonstrated good accuracy, differences in prediction, particularly with nonnative ligands can occur. While more efficient than biological experiments, MD simulations are computationally intense, expensive, and require the availability of 3D protein structures, which may be unavailable for many potential proteins of interest. In addition, MD simulations are sensitive to the positioning of the ligand within the target protein, requiring docking to the initial binding site which may be incorrect or not optimal.

Because MD simulations cannot be performed for many proteins due to the lack of confirmed 3D structures, the QSAR model was built using primary sequence-derived protein descriptors. These protein descriptors correspond to protein physical, structural, functional, and physiological characteristics.^33^ Once trained, their model relating protein descriptors with binding energies with PSMA-ligands could then be used to screen available genomic data of secretory and membrane proteins for which no published 3D protein structures are available, and/or those for which no molecular docking and molecular dynamics simulations can be performed. Yet sequence-derived data were not able to account for post-translational modifications, or changes in protein expression or metabolism in tissues. Despite this, the trained model was robust and performed sufficiently well upon the test dataset with an *R*^2^ of 0.7, demonstrating that the model could account for much of the diversity within the training dataset. While the predicted values correlated with MD-simulated values, there is a difference in quantitation with the predicted values being less negative (lower binding affinities) compared with the MD-simulated values (Fig. 1). The regressive nature of the QSAR model seemed to output the predictions along a smoothed trend. The feature descriptors describe global protein structure resemblance, which lacks markers based unique to the binding pocket. This allowed the model to distinguish between protein structures without having to manually distinguish a binding pocket, which allows for evaluation of potential protein–ligand interactions if binding occurs at a site different than the known binding site of a protein, or if a protein structure has no known ligand or defined binding pocket. Meanwhile, the model may struggle to distinguish between slight changes in amino acid composition, which could change enzymatic or binding properties.

Pairwise alignments of proteins were used to identify any potential targets from a highly selected group that had sufficient overlap with PSMA to have similar structural and binding capabilities toward the target ligands. Such alignments are often used for investigating shared or similar functionality of two proteins (Fig. 2).^45^ Alignments of proteins meeting criteria as targets and passing selectivity filters for the salivary gland demonstrated 3 proteins with significant overlap (5/7) with the PSMA binding pocket at residues E424, R463, N519, R534, and R536, corresponding to the binding pocket of PSMA.^46^ Importantly, these targets demonstrated overlap of key ‘arginine patch’ amino acids (R463, R534, and R536), which are believed to be crucial for interacting with the negative charged glutamate moieties of the native PSMA ligand to correctly orient the substrate in the binding pocket. The importance of these amino acids is demonstrated by their significant conservation across PSMA in mammalian and other species, as well as mutation analysis.

Testing with selected cell lines failed to demonstrate any significant PSMA uptake (Fig. 3), which ruled out possible contributions from glutamate receptors and transporters as well as folate receptors and transporters as off-targets corroborating with negative clinical trial results with monosodium glutamate and folic polyglutamate, respectively.^47,48^ However, *in vitro* cell assays are not optimal for testing secretary proteins, which are distributed in the vicinities of the cells and may be reabsorbed by the cell. Mice are a commonly used model for human salivary gland development, and limitations in their usage have been documented.^49–55^ Imaging with small animal PET (Fig. 4) using PSMA knockout (PSMA−/−) mice nonetheless demonstrated an overall reduced PSMA-radioligand uptake, including in the salivary glands, which might explain similar muscle to parotid uptake ratios in the wild-type and PSMA-knockout mice. There is a discrepancy between model-predicted and MD-simulated binding affinity of the PSMA-ligand to one of the PSMA (Naalad1 or GCP II) homology, Naalad2 (GCP III, see the bottom of Supplementary Table S3). Yet, they assessed the validity of GCP III as an off-target in the salivary gland before.^56^ The authors concluded that the uptake of PSMA ligands is still mediated through the existing PSMA in the salivary glands albeit through a different process than that in prostate cancer.

## References

[1] Sung H, Ferlay J, Siegel RL, et al. Global cancer statistics 2020: GLOBOCAN estimates of incidence and mortality worldwide for 36 cancers in 185 countries. CA Cancer J Clin 2021;71(3):209–249; doi: 10.3322/caac.21660 33538338

[2] Wüstemann T, Haberkorn U, Babich J, et al. Targeting prostate cancer: Prostate-specific membrane antigen based diagnosis and therapy. Med Res Rev 2019;39(1):40–69; doi: 10.1002/med.21508 29771460

[3] Kratochwil C, Bruchertseifer F, Giesel FL, et al. 225Ac-PSMA-617 for PSMA-targeted-radiation therapy of metastatic castration-resistant prostate cancer. J Nucl Med 2016;57(12):1941–1944; doi: 10.2967/jnumed.116.178673 27390158

[4] Heynickx N, Herrmann K, Vermeulen K, et al. The salivary glands as a dose limiting organ of PSMA—targeted radionuclide therapy: A review of the lessons learnt so far. Nucl Med Biol 2021;98–99:30–39; doi: 10.1016/j.nucmedbio.2021.04.003 34020337

[5] Gaertner FC, Halabi K, Ahmadzadehfar H, et al. Uptake of PSMA-ligands in normal tissues is dependent on tumor load in patients with prostate cancer. Oncotarget 2017;8(33):55094–55103; doi: 10.18632/oncotarget.19049 28903405 PMC5589644

[6] Silver DA, Pellicer I, Fair WR, et al. Prostate-specific membrane antigen expression in normal and malignant human tissues. Clin Cancer Res 1997;3(1):81–85. 9815541

[7] Mhawech‐Fauceglia P, Zhang S, Terracciano L, et al. Prostate‐specific membrane antigen (PSMA) protein expression in normal and neoplastic tissues and its sensitivity and specificity in prostate adenocarcinoma: An immunohistochemical study using mutiple tumour tissue microarray technique. Histopathology 2007;50(4):472–483; doi: 10.1111/j.1365-2559.2007.02635.x 17448023

[8] Rupp NJ, Umbricht CA, Pizzuto DA, et al. First Clinicopathologic evidence of a non–PSMA-related uptake mechanism for^68^ Ga-PSMA-11 in salivary glands. J Nucl Med 2019;60(9):1270–1276; doi: 10.2967/jnumed.118.222307 30737300

[9] Tönnesmann R, Meyer PT, Eder M, et al. [177Lu]Lu-PSMA-617 salivary gland uptake characterized by quantitative *In Vitro* autoradiography. Pharmaceuticals (Basel) 2019;12(1):18; doi: 10.3390/ph12010018 30678341 PMC6469177

[10] Gimeno A, Ojeda-Montes MJ, Tomás-Hernández S, et al. The light and dark sides of virtual screening: What is there to know? Int J Mol Sci 2019;20(6):1375; doi: 10.3390/ijms20061375 30893780 PMC6470506

[11] Maia EHB, Assis LC, de Oliveira TA, et al. Structure-based virtual screening: From classical to artificial intelligence. Front Chem 2020;8:343. Available from: https://www.frontiersin.org/articles/10.3389/fchem.2020.00343 [December 9, 2023]. 32411671 10.3389/fchem.2020.00343 PMC7200080

[12] Lavecchia A, Giovanni C. Virtual screening strategies in drug discovery: A critical review. Curr Med Chem 2013;20(23):2839–2860; doi: 10.2174/09298673113209990001 23651302

[13] Huang H, Zhang G, Zhou Y, et al. Reverse screening methods to search for the protein targets of chemopreventive compounds. Front Chem 2018;6:138. Available from: https://www.frontiersin.org/articles/10.3389/fchem.2018.00138 [December 9, 2023]. 29868550 10.3389/fchem.2018.00138 PMC5954125

[14] Deng Y, Roux B. Computations of standard binding free energies with molecular dynamics simulations. J Phys Chem B 2009;113(8):2234–2246; doi: 10.1021/jp807701h 19146384 PMC3837708

[15] Vilar S, Costanzi S. Predicting the biological activities through QSAR analysis and docking-based scoring. Methods Mol Biol 2012;914:271–284; doi: 10.1007/978-1-62703-023-6_16 22976034 PMC3445294

[16] Böselt L, Thürlemann M, Riniker S. Machine learning in QM/MM molecular dynamics simulations of condensed-phase systems. J Chem Theory Comput 2021;17(5):2641–2658; doi: 10.1021/acs.jctc.0c01112 33818085

[17] van der Gaag S, Bartelink IH, Vis AN, et al. Pharmacological optimization of PSMA-based radioligand therapy. Biomedicines 2022;10(12):3020; doi: 10.3390/biomedicines10123020 36551776 PMC9775864

[18] Petrov SA, Zyk NY, Machulkin AE, et al. PSMA-targeted low-molecular double conjugates for diagnostics and therapy. Eur J Med Chem 2021;225:113752; doi: 10.1016/j.ejmech.2021.113752 34464875

[19] Cardinale J, Schäfer M, Benešová M, et al. Preclinical Evaluation of 18 F-PSMA-1007, A new prostate-specific membrane antigen ligand for prostate cancer imaging. J Nucl Med 2017;58(3):425–431; doi: 10.2967/jnumed.116.181768 27789722

[20] Yang Y, Qin J, Liu H, et al. Molecular dynamics simulation, free energy calculation and structure-based 3D-QSAR studies of B-RAF kinase inhibitors. J Chem Inf Model 2011;51(3):680–692; doi: 10.1021/ci100427j 21338122

[21] Evans JC, Malhotra M, Cryan JF, et al. The therapeutic and diagnostic potential of the prostate specific membrane antigen/glutamate carboxypeptidase II (PSMA/GCPII) in cancer and neurological disease. Br J Pharmacol 2016;173(21):3041–3079; doi: 10.1111/bph.13576 27526115 PMC5056232

[22] Barinka C, Byun Y, Dusich CL, et al. Interactions between human glutamate carboxypeptidase II and urea-based inhibitors: Structural characterization. ^†^J Med Chem 2008;51(24):7737–7743; doi: 10.1021/jm800765e 19053759 PMC5516903

[23] Szabo Z, Mena E, Rowe SP, et al. Initial evaluation of [18F]DCFPyL for Prostate-Specific Membrane Antigen (PSMA)-targeted PET imaging of prostate cancer. Mol Imaging Biol 2015;17(4):565–574; doi: 10.1007/s11307-015-0850-8 25896814 PMC4531836

[24] Gaulton A, Bellis LJ, Bento AP, et al. ChEMBL: A large-scale bioactivity database for drug discovery. Nucleic Acids Res 2012;40(Database issue):D1100–D1107; doi: 10.1093/nar/gkr777 21948594 PMC3245175

[25] Berman HM, Westbrook J, Feng Z, et al. The protein data bank. Nucleic Acids Res 2000;28(1):235–242; doi: 10.1093/nar/28.1.235 10592235 PMC102472

[26] Macrae CF, Sovago I, Cottrell SJ, et al. *Mercury 4.0* : From visualization to analysis, design and prediction. J Appl Crystallogr 2020;53(Pt 1):226–235; doi: 10.1107/S1600576719014092 32047413 PMC6998782

[27] Jones G, Willett P, Glen RC, et al. Development and validation of a genetic algorithm for flexible docking11Edited by F. E. J Mol Biol 1997;267(3):727–748; doi: 10.1006/jmbi.1996.0897 9126849

[28] Korb O, Stützle T, Exner TE. Empirical scoring functions for advanced protein−ligand docking with plants. J Chem Inf Model 2009;49(1):84–96; doi: 10.1021/ci800298z 19125657

[29] Friesner RA, Banks JL, Murphy RB, et al. Glide: A new approach for rapid, accurate docking and scoring. 1. method and assessment of docking accuracy. J Med Chem 2004;47(7):1739–1749; doi: 10.1021/jm0306430 15027865

[30] Sousa da Silva AW, Vranken WF. ACPYPE—AnteChamber PYthon Parser interfacE. BMC Res Notes 2012;5(1):367; doi: 10.1186/1756-0500-5-367 22824207 PMC3461484

[31] Vanommeslaeghe K, MacKerell AD. Automation of the CHARMM general force field (CGenFF) I: Bond perception and atom typing. J Chem Inf Model 2012;52(12):3144–3154; doi: 10.1021/ci300363c 23146088 PMC3528824

[32] Valdés-Tresanco MS, Valdés-Tresanco ME, Valiente PA, et al. gmx_MMPBSA: A new tool to perform end-state free energy calculations with GROMACS. J Chem Theory Comput 2021;17(10):6281–6291; doi: 10.1021/acs.jctc.1c00645 34586825

[33] Cao DS, Xiao N, Xu QS, et al. Rcpi: R/Bioconductor package to generate various descriptors of proteins, compounds and their interactions. Bioinformatics 2015;31(2):279–281; doi: 10.1093/bioinformatics/btu624 25246429

[34] Chen Z, Zhao P, Li F, et al. iFeature: A python package and web server for features extraction and selection from protein and peptide sequences. Bioinformatics 2018;34(14):2499–2502; doi: 10.1093/bioinformatics/bty140 29528364 PMC6658705

[35] Uhlén M, Fagerberg L, Hallström BM, et al. Tissue-based map of the human proteome. Science 2015;347(6220):1260419; doi: 10.1126/science.1260419 25613900

[36] The UniProt Consortium. UniProt: The universal protein knowledgebase in 2023. Nucleic Acids Res 2023;51(D1):D523–D531; doi: 10.1093/nar/gkac1052 36408920 PMC9825514

[37] Needleman SB, Wunsch CD. A general method applicable to the search for similarities in the amino acid sequence of two proteins. J Mol Biol 1970;48(3):443–453; doi: 10.1016/0022-2836(70)90057-4 5420325

[38] Deng Y, Wang Y, Cherian C, et al. Synthesis and discovery of high affinity folate receptor-specific Glycinamide Ribonucleotide Formyltransferase inhibitors with antitumor activity. J Med Chem 2008;51(16):5052–5063; doi: 10.1021/jm8003366 18680275 PMC2748117

[39] Bacich DJ, Ramadan E, O’Keefe DS, et al. Deletion of the glutamate carboxypeptidase II gene in mice reveals a second enzyme activity that hydrolyzes N-acetylaspartylglutamate. J Neurochem 2002;83(1):20–29; doi: 10.1046/j.1471-4159.2002.01117.x 12358725

[40] Julyan PJ, Taylor JH, Hastings DL, et al. SUVpeak: A new parameter for quantification of uptake in FDG PET. Nucl Med Commun 2004;25(4). Available from: https://journals.lww.com/nuclearmedicinecomm/fulltext/2004/04000/suvpeak__a_new_parameter_for_quantification_of.40.aspx

[41] Fagerberg L, Hallström BM, Oksvold P, et al. Analysis of the Human Tissue-specific Expression by Genome-wide Integration of Transcriptomics and Antibody-based Proteomics. Mol Cell Proteomics 2014;13(2):397–406; doi: 10.1074/mcp.M113.035600 24309898 PMC3916642

[42] Heynickx N, Segers C, Coolkens A, et al. Characterization of non-specific uptake and retention mechanisms of [177Lu]Lu-PSMA-617 in the salivary glands. Pharmaceuticals (Basel) 2023;16(5):692; doi: 10.3390/ph16050692 37242475 PMC10224201

[43] Hotta M, Gafita A, Murthy V, et al. PSMA PET tumor–to–salivary gland ratio to predict response to [^177^ Lu]PSMA Radioligand therapy: An international multicenter retrospective study. J Nucl Med 2023;64(7):1024–1029; doi: 10.2967/jnumed.122.265242 36997329 PMC11937727

[44] Deshmukh DS, Madagi S, Savadatti V. Identification of potential anti-tumorigenic targets for rosemary components using dual reverse screening approaches. IJPBS 2013;3:399–408.

[45] Higdon R, Louie B, Kolker E. Modeling sequence and function similarity between proteins for protein functional annotation. Proc Int Symp High Perform Distrib Comput 2010;2010:499–502; doi: 10.1145/1851476.1851548 25101328 PMC4120521

[46] Davis MI, Bennett MJ, Thomas LM, et al. Crystal structure of prostate-specific membrane antigen, a tumor marker and peptidase. Proc Natl Acad Sci U S A 2005;102(17):5981–5986; doi: 10.1073/pnas.0502101102 15837926 PMC556220

[47] Harsini S, Saprunoff H, Alden T, et al. The effects of monosodium glutamate on PSMA radiotracer uptake in men with recurrent prostate cancer: A prospective, randomized, double-blind, placebo-controlled intraindividual imaging study. J Nucl Med 2021;62(1):81–87; doi: 10.2967/jnumed.120.246983 32385167

[48] Paganelli G, Sarnelli A, Severi S, et al. Dosimetry and safety of 177Lu PSMA-617 along with polyglutamate parotid gland protector: Preliminary results in metastatic castration-resistant prostate cancer patients. Eur J Nucl Med Mol Imaging 2020;47(13):3008–3017; doi: 10.1007/s00259-020-04856-1 32430583

[49] Musselmann K, Green JA, Sone K, et al. Salivary gland gene expression atlas identifies a new regulator of branching morphogenesis. J Dent Res 2011;90(9):1078–1084; doi: 10.1177/0022034511413131 21709141 PMC3318080

[50] Kondo Y, Nakamoto T, Jaramillo Y, et al. Functional differences in the acinar cells of the murine major salivary glands. J Dent Res 2015;94(5):715–721; doi: 10.1177/0022034515570943 25680367 PMC4502782

[51] Gluck C, Min S, Oyelakin A, et al. RNA-seq based transcriptomic map reveals new insights into mouse salivary gland development and maturation. BMC Genomics 2016;17(1):923; doi: 10.1186/s12864-016-3228-7 27852218 PMC5112738

[52] Knedlík T, Vorlová B, Navrátil V, et al. Mouse glutamate carboxypeptidase II (GCPII) has a similar enzyme activity and inhibition profile but a different tissue distribution to human GCPII. FEBS Open Bio 2017;7(9):1362–1378; doi: 10.1002/2211-5463.12276 PMC5586342 28904865

[53] Song EAC, Min S, Oyelakin A, et al. Genetic and scRNA-seq analysis reveals distinct cell populations that contribute to salivary gland development and maintenance. Sci Rep 2018;8(1):14043; doi: 10.1038/s41598-018-32343-z 30232460 PMC6145895

[54] Roy J, Warner BM, Basuli F, et al. Comparison of prostate-specific membrane antigen expression levels in human salivary glands to non-human primates and rodents. Cancer Biother Radiopharm 2020;35(4):284–291; doi: 10.1089/cbr.2019.3079 32074455 PMC7247045

[55] Simons BW, Turtle NF, Ulmert DH, et al. PSMA expression in the Hi‐Myc model; extended utility of a representative model of prostate adenocarcinoma for biological insight and as a drug discovery tool. Prostate 2019;79(6):678–685; doi: 10.1002/pros.23770 30656716 PMC6519119

[56] Lee Z, Heston WD, Wang X, et al. GCP III is not the “off-target” for urea-based PSMA ligands. Eur J Nucl Med Mol Imaging 2023;50(10):2944–2946; doi: 10.1007/s00259-023-06265-6 37191680 PMC10382371

