# Supplementary material for: Searching for Protein Off-Targets of Prostate-Specific Membrane Antigen-Targeting Radioligands in the Salivary Glands
Source: Cancer Biother Radiopharm. 2024 Dec 4;39(10):721–32. doi: 10.1089/cbr.2024.0066 (PMC11824224; doi:10.1089/cbr.2024.0066)
Supplement: Supplementary Data S1 [file cbr.2024.0066_supp_data_s1.pdf]

Supplementary information to “Searching for protein off-targets of PSMA-targeting radioligands in the salivary glands”

William Julian[1], Olga Sergeeva[1*], Wei Cao[1], Chunying Wu[1], Bernadette Erokwu[1], Christopher Flask[1], Lifang Zhang[1], Xinning Wang[2,1], James Basilion[1,2], Sichun Yang[3], Zhenghong Lee[1,2]

1. Radiology Department, Case Western Reserve University, Cleveland, OH, USA
2. Biomedical Engineering Department, Case Western Reserve University, Cleveland, OH, USA
3. Nutrition Department, Case Western Reserve University, Cleveland, OH, USA
*: Currently with Department of Surgery, Cleveland Clinic, Cleveland, OH, USA

Methods

Target ligands:

2-PMPA, MUD, and DCFPyL were used as the target ligands. 2-PMPA, also known as 2-(phosphonomethyl)pentanedioic acid, is phosphonate-based derivative of glutamate originally designed as an GCP II inhibitor.[1] MUD, known as (S)-2-(3-((R)-1-carboxy-2-methylthio)ethyl)ureido)pentanedioic acid or DCMC, is a urea based GCPII inhibitor.[2] DCFPyL, also known as 2-(3-(1-carboxy-5-[(6-[18F]fluoro-pyridine-3-carbonyl)-amino]-pentyl)-ureido)-pentanedioic acid, is a second generation urea-based GCPII ligand.[3] Ligand structures were obtained from the RCSB and ChemBL databases.[4,5]

Unknown Targets

Protein structures for all target proteins were obtained from the RCSB database.

Molecular docking

Molecular docking was performed prior to molecular dynamics simulations to position ligands within the protein. 2-PMPA, MUD, and DCFPyL were selected for molecular docking with target proteins. Chemical structures of ligands were retrieved from RCSB and ChEMBL databases and prepared using CCDC Mercury software.[5,6]

Docking was performed using the GOLD software package with default parameters.[7] The GOLD algorithm employs a genetic algorithm to explore the conformational space of ligands within the

predefined receptor grid. The top-ranked pose represented by scoring function were chosen for each of the 3 ligands for usage in molecular dynamics simulations.

The ChemPLP scoring function was employed to evaluate the binding affinity of ligands within the binding site.[8] The ChemPLP is a combination of piecewise linear potential combined with torsional, hydrogen bonding and metal coefficients. The top-ranked ligand poses were visually inspected to ensure proper orientation within the binding site. Swiss PDB-viewer was used for the addition of missing protein residues after docking was completed.

Target protein structures for docking were obtained from the RCSB protein database and prepared by removing water molecules and adding hydrogen atoms using GOLD or Schrödinger-2023-3.[9] The active site, including the pocket comprising of E272, N379, R389, R387, R319, was defined based on literature review and/or the positioning of external ligands within the protein structure.

Molecular dynamics (MD) simulations

Protein-ligand complexes were obtained following molecular docking. Protein-ligand complexes were simulated using 4 repeats with both Charmm36 and Amber forcefields. The ligand topology and parameter files were generated using the acpype parameterization tool for Amber forcefields, and CgenFF tool was used for parameterization of the ligand for Charmm forcefields. [10,11]

The protein-ligand complex was solvated in a dodecahedral periodic box with TIP3P water molecules with a box size proportional to the max diameter of the complex. Appropriate counterions were added to maintain system neutrality to a concentration of 0.15M. The system was energy-minimized to remove steric clashes and achieve a stable starting structure. The prepared system was subjected to energy minimization using the steepest descent algorithm until convergence was achieved, ensuring a force tolerance below 1000 kJ mol^{-1} nm^{-1}.

The system was equilibrated in the canonical (NVT) and isothermal-isobaric (NPT) methods to stabilize temperature and pressure, respectively. The Berendsen thermostat and Parrinello-Rahman barostat were employed during these equilibration steps. A MD simulation was performed using the leap-frog integration scheme with a time step of 2 fs. Simulations were performed for 10ns.

The CHARMM36 and Amber force fields were utilized for the protein, and ligand parameters were retained from the setup phase. Non-bonded interactions were treated with a cutoff of 1.2 nm, and long-range electrostatics were computed using the Particle Mesh Ewald (PME) method. The system was maintained at a constant temperature of 300 K using the modified Berendsen thermostat and a pressure of 1 bar using the Parrinello-Rahman barostat. Coordinates, velocities, and energies were saved at every 50 ps during the production run for subsequent analysis.

The free energy of binding (ΔG_bind) was calculated using the GMX_MMPBSA tool. [12] Free energy of binding was calculated as the sum of the molecular mechanics energy (ΔE_MM), solvation free energy (ΔG_solv), and entropy contribution (−TΔS) for the given protein ligand complex. All calculations were performed using generalized born parameters.

All MD calculations and free energy calculations were performed on the High-Performance Computing Resource in the Core Facility for Advanced Research Computing at Case Western Reserve University. Docking simulations were performed on Intel i7-10700 CPU processor.

QSAR

Protein-ligand activity for QS***A***R modeling was obtained from the output of free energy of binding calculations following MD simulations. This data included a set of selected proteins, 3 ligands, and their associated experimental free energies of binding.

Structure data for Q***S***AR modeling came from sequence data for protein-ligand pairs, for each of which a set of descriptor values was calculated. Ligand descriptors were calculated using the rCDK package using the R software, resulting in each of the 3 ligands having 197 descriptors consisted of physicochemical properties, graph theoretical indices and functional group counts.[13] Protein descriptors were calculated using the iFeature program, resulting in each protein being characterized with 13494 descriptors including amino acid composition, dipeptide composition, autocorrelation descriptors, quasi-sequence order, amphiphilic pseudo amino acid composition, and total amino acid properties, etc. [14] (The two long lists of descriptors enumerating ligand and protein structural features, respectively, are available

upon request.) Proteins under 30 AA in length were excluded from analysis given the constraints of protein characterization requiring sequence length > 30 aa.

Each resulting protein-ligand pair comprising the concatenated protein-ligand descriptors underwent principal component analysis (PCA) to reduce data dimensionality. The resulting dataset with selected PCA components was randomly split into training (75% of the data) and testing sets (25 % of the data) to evaluate model performance.

QSAR models were built using the tidymodels framework in R. The model formula was defined with the dependent variable (binding energy) and independent variables (molecular descriptors). Different models, such as linear regression, decision trees, or random forests, were considered based on the nature of the data. Data preprocessing steps, such as centering and scaling of numerical predictors, were applied to ensure numerical stability and comparability.

R-squared and RMSE (Root Mean Squared Error) were used to evaluate the performance of the model on the test data set. R version 4.3.2 was used to conduct the analysis.

Library Screening

Protein libraries from the human protein atlas and Uniprot databases comprising secreted and membrane proteins were downloaded and screened using the model following characterization using the methods described previously. [15,16] 2-PMPA was used as the screening ligand. Proteins with binding energies in the top 25% of screened proteins were designated target candidates and subject to cross-validation with databases of salivary protein expression. High or selective expression was defined by having 4-fold higher expression in the salivary gland compared to other tissues, expression limited to the salivary gland or expression in the salivary gland and less than 1/3 of human tissues. Proteins identified by the model as targets and meeting expression criteria were selected for further analysis via pairwise protein alignments.

Pairwise Protein-Alignments

Following protein screening, proteins designated as potential targets were compared with databases of proteins known to be selectively or highly expressed in the salivary gland relative to other

tissues. Proteins designated as targets found to be highly or selectively expressed in the salivary gland were subjected to pairwise alignment using the EMBL-EBI pairwise sequence alignment tool. [17] Sequences were compared against the FOLH1 protein sequence at 18 separate amino acids comprising 4 major structural features of the PSMA binding pocket. Sequences with >60% sequences homology at any of the 4 major structural features were simulated using MD simulations as described earlier.

Cellular/Animal Models

In-vitro studies were carried out to test PSMA-ligand binding to possible non-PSMA protein targets used in the training set. Cell lines, from ATCC, with low PSMA expression were selected for ligand binding assays, and possible non-PSMA targets with high expression in each cell line are listed in **Table I**. The RT16 and D4 cell lines, which were engineered from the parent R2 (CHO) cell lines to express human folate receptors α and ß, respectively, were generously given by Dr. Larry H. Matherly from Karmanos Cancer Institute at Wayne State University. [18]

For each cell line, a half million cells were incubated with different amounts (3.125 nM to 100nM) of H-3 labeled simplest PSMA radioligand (S)-2-(3-((S)-5-amino-1-carboxypentyl)ureido)pentanedioic acid ([3H]ZJ-24, RC TRITEC AG, Teufen, Switzerland) in 50 mM Tris, pH 7.5 in the range of tracer dose for 60 min followed by washing, palleting, and liquid-scintillation counting to measure total uptake; the other portions had 10 μM cold PSMA-ligand ZJ-24 added for measuring non-specific uptake via competition. Cells were incubated at 37 ^{0}C for 1 hour. A subtraction yielded specific uptake.

In-vivo small animal PET imaging using the clinical ligand [68Ga]Ga-PSMA-11 was performed with 3 PSMA null (PSMA-/-) mice, in which PSMA expression is disabled and compared with 3 wildtype (wt) mice for radioligand uptake in the salivary glands.[19] The PSMA knock out mice were identified by genotyping via PCR analysis of tail DNA samples. Three primers were used for genotyping, including the Folh1 IntA primer: 5’-ATTCAATCCTGCTCAGACCC-3’, (2) Folh1 S49 primer: 5’-gtagaagagaactgctgagga- 3’, and (3)Neomycin S primer: 5’-AGCAGGCATGCTGGGGATGC-3’. The

primers Folh1 IntA and Folh1 S49 were used to amplify the endogenous gene. Folh1 S49 and Neomycin S primers identified the PSMA knockout gene. PCR was performed at 95°C for 15 s, 58°C for 30 s and 72°C for 1 min, for 35 cycles. PCR products were analyzed by electrophoresis with 2% Agarose gel. GelRed DNA stain was used to identify DNA bands on the gel. Mouse scans were performed on 9.4T Bruker Biospec preclinical MRI scanner (Bruker Corp., Billerica, MA, USA). Each mouse was anesthetized with isoflurane and positioned within a Cubresa NuPET PET insert (Winnipeg, MB, Canada) and PET-MRI compatible radiofrequency volume MRI coil (ID = 35 mm). [20] 200 μCi (7.4 MBq) of the radioligand [68Ga]Ga-PSMA-11 was injected intravenously via tail vein. 5-min static small animal PET scans were acquired with the NuPET insert at 0.5 and 1.0 hour post-injection (some animals only had 1-hour post-injection scans). Following initial localizer scans, mouse MRI images were acquired simultaneously with a coronal 3D True FISP (Fast Imaging with Steady-state Free Precession) acquisition (FOV = 60 x 30 x 30mm, matrix size = 256 x 128 x 128, TR/TE = 4.0/2.0 ms, flip angle = 30 degrees, 5 signal averages, scan time = 6 minutes) using the reconstruction method on the system supplied by Bruker. After scanning, MR and PET scans were aligned for region definition and quantification, i.e., calculation of region-based standardized uptake values (SUVs) such as SUVmax and SUVpeak. [21]

References

1. Evans JC, Malhotra M, Cryan JF, O'Driscoll CM. The therapeutic and diagnostic potential of the prostate specific membrane antigen/glutamate carboxypeptidase II (PSMA/GCPII) in cancer and neurological disease. *Br J Pharmacol*. 2016;173(21):3041-3079. doi:10.1111/bph.13576

2. Barinka C, Byun Y, Dusich CL, et al. Interactions between Human Glutamate Carboxypeptidase II and Urea-Based Inhibitors: Structural Characterization [†]. *J Med Chem*. 2008;51(24):7737-7743. doi:10.1021/jm800765e

3. Szabo Z, Mena E, Rowe SP, et al. Initial Evaluation of [18F]DCFPyL for Prostate-Specific Membrane Antigen (PSMA)-Targeted PET Imaging of Prostate Cancer. *Mol Imaging Biol MIB Off Publ Acad Mol Imaging*. 2015;17(4):565-574. doi:10.1007/s11307-015-0850-8

4. Gaulton A, Bellis LJ, Bento AP, et al. ChEMBL: a large-scale bioactivity database for drug discovery. *Nucleic Acids Res*. 2012;40(Database issue):D1100-D1107. doi:10.1093/nar/gkr777

5. Berman HM. The Protein Data Bank. *Nucleic Acids Res*. 2000;28(1):235-242. doi:10.1093/nar/28.1.235

6. Macrae CF, Sovago I, Cottrell SJ, et al. *Mercury 4.0* : from visualization to analysis, design and prediction. *J Appl Crystallogr*. 2020;53(1):226-235. doi:10.1107/S1600576719014092

7. Jones G, Willett P, Glen RC, Leach AR, Taylor R. Development and validation of a genetic algorithm for flexible docking11Edited by F. E. Cohen. *J Mol Biol*. 1997;267(3):727-748. doi:10.1006/jmbi.1996.0897

8. Korb O, Stützle T, Exner TE. Empirical Scoring Functions for Advanced Protein−Ligand Docking with PLANTS. *J Chem Inf Model*. 2009;49(1):84-96. doi:10.1021/ci800298z

9. Friesner RA, Banks JL, Murphy RB, et al. Glide: A New Approach for Rapid, Accurate Docking and Scoring. 1. Method and Assessment of Docking Accuracy. *J Med Chem*. 2004;47(7):1739-1749. doi:10.1021/jm0306430

10. Sousa da Silva AW, Vranken WF. ACPYPE - AnteChamber PYthon Parser interfacE. *BMC Res Notes*. 2012;5(1):367. doi:10.1186/1756-0500-5-367

11. Vanommeslaeghe K, MacKerell AD. Automation of the CHARMM General Force Field (CGenFF) I: bond perception and atom typing. *J Chem Inf Model*. 2012;52(12):3144-3154. doi:10.1021/ci300363c

12. Valdés-Tresanco MS, Valdés-Tresanco ME, Valiente PA, Moreno E. gmx_MMPBSA: A New Tool to Perform End-State Free Energy Calculations with GROMACS. *J Chem Theory Comput*. 2021;17(10):6281-6291. doi:10.1021/acs.jctc.1c00645

13. Cao DS, Xiao N, Xu QS, Chen AF. Rcpi: R/Bioconductor package to generate various descriptors of proteins, compounds and their interactions. *Bioinformatics*. 2015;31(2):279-281. doi:10.1093/bioinformatics/btu624

14. Chen Z, Zhao P, Li F, et al. iFeature: a Python package and web server for features extraction and selection from protein and peptide sequences. *Bioinformatics*. 2018;34(14):2499-2502. doi:10.1093/bioinformatics/bty140

15. Uhlén M, Fagerberg L, Hallström BM, et al. Tissue-based map of the human proteome. *Science*. 2015;347(6220):1260419. doi:10.1126/science.1260419

16. The UniProt Consortium. UniProt: the Universal Protein Knowledgebase in 2023. *Nucleic Acids Res*. 2023;51(D1):D523-D531. doi:10.1093/nar/gkac1052

17. Needleman SB, Wunsch CD. A general method applicable to the search for similarities in the amino acid sequence of two proteins. *J Mol Biol*. 1970;48(3):443-453. doi:10.1016/0022-2836(70)90057-4

18. Deng Y, Wang Y, Cherian C, et al. Synthesis and Discovery of High Affinity Folate Receptor-Specific Glycinamide Ribonucleotide Formyltransferase Inhibitors With Antitumor Activity. *J Med Chem*. 2008;51(16):5052-5063. doi:10.1021/jm8003366

19. Bacich DJ, Ramadan E, O'Keefe DS, et al. Deletion of the glutamate carboxypeptidase II gene in mice reveals a second enzyme activity that hydrolyzes N-acetylaspartylglutamate. *J Neurochem*. 2002;83(1):20-29. doi:10.1046/j.1471-4159.2002.01117.x

20. Pollard AC, de la Cerda J, Schuler FW, Kingsley CV, Gammon ST, Pagel MD. Evaluations of the performances of PET and MRI in a simultaneous PET/MRI instrument for pre-clinical imaging. *EJNMMI Phys*. 2022;9(1):70. doi:10.1186/s40658-022-00483-x

21. Julyan PJ, Taylor JH, Hastings DL, Williams HA, Zweit J. SUVpeak: a new parameter for quantification of uptake in FDG PET. *Nucl Med Commun*. 2004;25(4). https://journals.lww.com/nuclearmedicinecomm/fulltext/2004/04000/suvpeak__a_new_parameter_for_quantification_of.40.aspx
