# Supplementary material for: Searching for Protein Off-Targets of Prostate-Specific Membrane Antigen-Targeting Radioligands in the Salivary Glands
Source: Cancer Biother Radiopharm. 2024 Dec 4;39(10):721–32. doi: 10.1089/cbr.2024.0066 (PMC11824224; doi:10.1089/cbr.2024.0066)
Supplement: Supplementary Table S1 [file cbr.2024.0066_supp_tables.pdf]

Supplementary Table 1. Free energies of binding (Kj/mol) between three know PSMA-ligands and target candidates generated by molecular dynamics simulations and used in QSAR model training.

Gene Name	2PMPA	MUD	DCPYL
AAK1	-13.4213	-17.3063	-36.6763
ABCC1	-4.56	-7.74	-10.5825
ABCC6	-6.14	-7.785	-19.6625
ACP3	-14.0983	-15.7538	-30.5945
ACVR1	-12.28	-19.275	-26.9258
ACVR2A	-15.7413	-14.2725	-21.6363
ADAM10	-15.655	-21.2588	-38
ADAM17	-10.0883	-24.05	-21.5933
ADAMTS1	-10.265	-20.8075	-29.18
ADIPOR1	-23.99	-38.42	-44.27
ADIPOR2	-17.605	-32.8042	-46.015
CALCRL	-9.79	-15.5983	-24.0858
CASR	-8.35875	-14.885	-15.5763
CCKAR	-11.3025	-23.7675	-33.625
CCR2	-6.0575	-10.155	-15.2975
CCR5	-13.8838	-19.4825	-20.6625
CD81	-1.905	-9.355	-12.5675
CFTR	-6.79625	-16.335	-21.355
CHRM1	-19.1063	-31.51	-39.7975
CHRNA7	-15.4713	-23.4825	-30.4525
CLH17	-5.025	-15.6113	-16.62
CNR1	-14.98	-27.9	-36.8488
CPA1	-20.7188	-33.2125	-34.4938
CPB1	-37.0175	-37.74	-45.3425
CPB2	-22.0233	-35.5792	-43.19
CPM	-4.435	-6.7575	-32.4325
CPTP	-4.8375	-12.305	-34.325
CXCR4	-10.3238	-12.3025	-18.5525
DLG4	-6.175	-9.7775	-16.0225
DRD2	-14.1225	-21.125	-26.3563
DRD3	-18.6325	-23.825	-25.6925
DUOX1	-7.70875	-16.5888	-27.8088
EDN3	-8.14	-13.7475	-22.4725
EDNRB	-5.7225	-12.6875	-23.535
ENPEP	-15.4975	-27.245	-22.0725
EPHB2	-8.3275	-12.2525	-19.055
FAM20A	-11.6925	-18.4075	-30.53
FFAR1	-19.475	-32.4	-40.8125
FOLR1	-21.91	-38.48	-24.57
FURIN	-6.44	-12.9775	-7.4275
GABBR1	-11.0725	-12.13	-13.2275
GCPii	-24.61	-33.0567	-45.3667
GLP1	-1.635	-4.3225	-6.31
GRIA1	-6.385	-13.995	-16.5275
GRIA2	-22.56	-39.8825	-44.505
GRIK1	-25.3525	-24.3825	-28.4575

GRIK2	-28.355	-27.3825	-24.255
GRIN1	-22.6025	-25.9825	-25.515
GRIN2A	-38.205	-45.9425	-38.9925
GRIN2B	-5.7675	-12.14	-15.5575
INSR	-12.71	-22.2225	-26.39
ITGAL	-13.715	-15.94	-19.29
KDR	-11.1625	-16.5975	-27.98
LCN2	-2.1425	-7.6175	-12.9075
LNPEP	-17.86	-11.0475	-8.77333
LTB4R	-18.655	-21.2175	-28.4
LTF	-41.535	-37.9375	-41.41
MELTF	-20.635	-35.6625	-39.9975
MMP13	-7.5475	-17.935	-19.65
MMP3	-16.18	-14.455	-30.31
MMP7	-11.27	-23.87	-19.6475
MTNR1A	-2.5675	-9.2175	-6.9225
NAALADL1	-2.52	-26.42	-36.58
NPY1R	-13.22	-23.715	-26.725
NTRK2	-10.4	-25.175	-25.2525
OSTM1	-4.4675	-12.075	-5.905
PTGER3	-17.54	-35.1125	-44.395
PTH1R	-9.5875	-4.2425	-8.7425
PTK2B	-17.8975	-20.125	-25.04
SIGMAR1	-20.9983	-33.6317	-46.085
SLC9A1	-13.545	-15.94	-23.605
SMO	-19.0683	-22.67	-31.2283
SORT1	-12.4045	-16.9118	-19.891
TACR1	-10.1325	-18.862	-23.37
TCN1	-8.63	-11.01	-17.1338
TFRC	-6.155	-10.8667	-2.33
TMPRSS15	-7.87	-13.95	-9.145
TRPV1	-9.32571	-21.0171	-25.604
ZG16B	-3.1475	-5.36333	-8.55

Supplementary Table 2. Target proteins selected by QSAR model with high or selective salivary expression with model predicted binding affinity to PSMA-ligands and RNA expression data for salivary glands.

Uniprot ID	Gene name	Model Prediction	RNA tissue specificity score*	Tissue RNA - esophagus [nTPM]*	Tissue RNA - salivary gland [nTPM]*
ACM1_HUMAN	CHRM1	-28.2227	10	0.3	28
ACM3_HUMAN	CHRM3	-16.9035		7.2	17
AMTN_HUMAN	AMTN	-16.9874		0.4	6.6
AMY1A_HUMAN	AMY1A	-17.1482	308	0.2	8032.9
AMY1B_HUMAN	AMY1B	-17.1482	97	0.2	10157.7
AMY1C_HUMAN	AMY1C	-17.1482	66	0.2	8146.9
C16L2_HUMAN	CD164L2	-19.7791		19.4	13.1
C1QT3_HUMAN	C1QTNF3	-18.8232		32.1	50.7
CCL28_HUMAN	CCL28	-23.3682	5	3.9	292
CE046_HUMAN	C5orf46	-18.7734	8	0.3	176.1
CEAM6_HUMAN	CEACAM6	-16.5694		189.8	172.4
CHP3_HUMAN	TESC	-19.7741		3.2	320.1
CHST9_HUMAN	CHST9	-16.9561		1.6	13.4
CLD17_HUMAN	CLDN17	-17.9398	4	6	6.2
CXCL5_HUMAN	CXCL5	-17.7754	4	0.1	48.7
CXL17_HUMAN	CXCL17	-23.1966	4	117.2	141.3
CYTD_HUMAN	CST5	-17.3798	961	0.4	2090.3
CYTN_HUMAN	CST1	-16.652	120	0.1	27534.6
CYTS_HUMAN	CST4	-16.6054	516	0	28148.8
CYTT_HUMAN	CST2	-17.3344	638	0	5804.2
D103A_HUMAN	DEFB103A	-23.0291		2.2	3.1
DEFB1_HUMAN	DEFB1	-22.4148	5	185.3	1917.1
DNS2B_HUMAN	DNASE2B	-17.2807	25	0	165.3
ELAP1_HUMAN	ELAPOR1	-17.216		1.5	161.2
FAM3D_HUMAN	FAM3D	-18.0778	5	257.4	454.7
FBN3_HUMAN	FBN3	-20.2996		0.2	3.9
FDSCP_HUMAN	FDCSP	-20.6913	27	13	6415.4
GDF5_HUMAN	GDF5	-19.2643	4	0	9.6
GGTA1_HUMAN	GGTA1	-17.0391		23.4	129.2
HIS1_HUMAN	HTN1	-23.889	2616	0	47123.7
HIS3_HUMAN	HTN3	-24.1618	1103	0.1	131918.2
HV146_HUMAN	IGHV1-46	-16.9151	4	4.3	20.2
HV335_HUMAN	IGHV3-35	-20.0181	4	0.5	7.3
HV374_HUMAN	IGHV3-74	-19.1693	6	84.4	73.5
HV428_HUMAN	IGHV4-28	-19.2442	5	12.1	38.5
HV692_HUMAN	IGHV1-69-2	-17.4364	5	2	15.8
IGFL1_HUMAN	IGFL1	-20.2029	9	109.8	33.5
INVO_HUMAN	IVL	-18.4678	5	289.9	91.7
ISK6_HUMAN	SPINK6	-23.2195		2.3	6.7
KLK12_HUMAN	KLK12	-17.8356	12	202.8	62.8
KLK15_HUMAN	KLK15	-17.0788		0	3.9
KV37_HUMAN	IGKV3-7	-18.1285		1.5	15.4
KVD15_HUMAN	IGKV3D-15	-16.938	6	15.8	52.2

LJ01_HUMAN	IGLJ1	-23.6396	5	1.9	33.2
LRC26_HUMAN	LRRC26	-16.9093	5	2.7	281.5
LV233_HUMAN	IGLV2-33	-18.3753		0.2	2.5
LV545_HUMAN	IGLV5-45	-16.608		39.1	8.3
LVX54_HUMAN	IGLV10-54	-18.4157	7	21.7	245.2
LYSC_HUMAN	LYZ	-21.6011		102.7	20501.5
MIA_HUMAN	MIA	-18.8535		6.4	41.9
MMP3_HUMAN	MMP3	-16.6238		0.7	277.6
MUC16_HUMAN	MUC16	-18.5246	4	0.3	5.9
MUC5B_HUMAN	MUC5B	-18.6528	12	98.1	2543.7
MUC7_HUMAN	MUC7	-20.459	877	2	25265.8
NCMAP_HUMAN	NCMAP	-17.7422		0.2	7.6
NUCB2_HUMAN	NUCB2	-17.574		90.3	493.3
ODAM_HUMAN	ODAM	-17.24	37	0.2	360.6
PIP_HUMAN	PIP	-18.7327	5	1.3	16308.1
PLPP2_HUMAN	PLPP2	-16.6873		26.5	102.6
PRB2_HUMAN	PRB2	-21.8258	3818	0	11778.1
PRB3_HUMAN	PRB3	-20.3694	1882	0.1	13472.2
PROL1_HUMAN	OPRPN	-17.3129	5	0	142.3
PROL4_HUMAN	PRR4	-18.2999	45	143.9	6424.4
PRP1_HUMAN	PRB1	-22.0819	992	0	6182.2
PRPC_HUMAN	PRH2	-18.8787	909	18.7	22313.4
PRR27_HUMAN	PRR27	-17.0815	959	0.1	1071.7
RNAS8_HUMAN	RNASE8	-17.4377	229	0	53.2
SG1C1_HUMAN	SCGB1C1	-18.9893		0.1	1
SG3A1_HUMAN	SCGB3A1	-16.7047	4	72.7	724.7
SLPI_HUMAN	SLPI	-23.4843	7	1334.2	13729.2
SMR3A_HUMAN	SMR3A	-20.7493	59	0.1	58
SMR3B_HUMAN	SMR3B	-20.4341	948	8.6	210537.1
STAT_HUMAN	STATH	-18.9491	925	2.6	8851
TACD2_HUMAN	TACSTD2	-17.1923		961.9	454.7
TFF3_HUMAN	TFF3	-22.1513		32.9	330.1
TPD53_HUMAN	TPD52L1	-18.6536		148.1	289.9
TRFL_HUMAN	LTF	-32.8224	4	22.3	1128.6
TRFM_HUMAN	MELTF	-23.7041		2.8	69.2
VWDE_HUMAN	VWDE	-16.7615		0.1	2.9
WFDC2_HUMAN	WFDC2	-18.7862		53.1	1926.5
WNT5A_HUMAN	WNT5A	-17.0151		16.5	33.5

* Data obtained from Mathias Uhlén et al. ,Tissue-based map of the human proteome.Science347,1260419(2015).DOI:10.1126/science.1260419

Supplementary Table 3. Simulated (molecular dynamics) and predicted (QSAR model) free-energies of binding for glutamate receptors and transporters (orange), folate receptors and transporters (blue), and PSMA homologs (red).

Gene Name	Uniprot ID	3D model	Enzymatic activity	Simulation Free Energy	Model Prediction
GRIN2D	NMDE4_HUMAN	Yes	Yes	Not simulated	-13.74
GRID2	GRID2_HUMAN	Yes	Yes	Not simulated	-12.92
GRIN3B	NMD3B_HUMAN	No	Yes	Not simulated	-14.17
GRIK1	GRIK1_HUMAN	Yes	Yes	-25.3525	-22.27
GRIA1	GRIA1_HUMAN	Yes	Yes	-6.385	-16.15
GRIA2	GRIA2_HUMAN	Yes	Yes	-22.56	-24.94
GRIA3	GRIA3_HUMAN	No	Yes	Not simulated	-17.93
SLC1A4	SATT_HUMAN	Yes	Yes	Not simulated	-13.70
GRIA4	GRIA4_HUMAN	No	Yes	Not simulated	-17.39
GRIN2A	NMDE1_HUMAN	Yes	Yes	-38.205	-26.82
GRIK2	GRIK2_HUMAN	Yes	Yes	-28.355	-20.96
GRIK3	GRIK3_HUMAN	No	Yes	Not simulated	-18.14
GRIN2B	NMDE2_HUMAN	Yes	Yes	-5.7675	-9.55
GRIN2C	NMDE3_HUMAN	Yes	Yes	Not simulated	-12.82
GRIK4	GRIK4_HUMAN	No	Yes	Not simulated	-13.43
GRIK5	GRIK5_HUMAN	No	Yes	Not simulated	-13.50
SLC17A8	VGLU3_HUMAN	No	Yes	Not simulated	-13.29
GRIN3A	NMD3A_HUMAN	No	Yes	Not simulated	-13.95
SLC17A7	VGLU1_HUMAN	No	Yes	Not simulated	-13.38
SLC17A6	VGLU2_HUMAN	No	Yes	Not simulated	-13.34
GRID1	GRID1_HUMAN	No	Yes	Not simulated	-13.21
GRM8	GRM8_HUMAN	Yes	Yes	Not simulated	-13.07
SLC1A7	EAA5_HUMAN	No	Yes	Not simulated	-14.56
GRM6	GRM6_HUMAN	No	Yes	Not simulated	-13.52
SLC25A12	S2512_HUMAN	Yes	Yes	Not simulated	-14.47
GRM5	GRM5_HUMAN	Yes	Yes	Not simulated	-13.13
SLC1A3	EAA1_HUMAN	Yes	Yes	Not simulated	-13.98
SLC1A2	EAA2_HUMAN	Yes	Yes	-23.69875	-14.68
SLC1A1	EAA3_HUMAN	Yes	Yes	-23.905	-14.93
SLC1A6	EAA4_HUMAN	No	Yes	Not simulated	-15.00
GRIN1	NMDZ1_HUMAN	Yes	Yes	-22.6025	-13.88
GRM1	GRM1_HUMAN	Yes	Yes	Not simulated	-13.15
GRM2	GRM2_HUMAN	Yes	Yes	Not simulated	-14.56
GRM7	GRM7_HUMAN	Yes	Yes	Not simulated	-14.06
GRM3	GRM3_HUMAN	Yes	Yes	Not simulated	-13.45
GRM4	GRM4_HUMAN	Yes	Yes	Not simulated	-13.99
SLC38A6	S38A6_HUMAN	No	Yes	Not simulated	-13.21
SLC25A18	GHC2_HUMAN	No	Yes	Not simulated	-15.63
SLC25A22	GHC1_HUMAN	No	Yes	Not simulated	-15.16
SLC25A13	S2513_HUMAN	Yes	Yes	Not simulated	-16.09
SLC7A11	XCT_HUMAN	Yes	Yes	-5.33375	-13.50
SLC46A1	PCFT_HUMAN	No	Yes	Not simulated	-14.46
IZUMO1R	JUNO_HUMAN	Yes	Non-folate binding	Not simulated	-15.89
FTCD	FTCD_HUMAN	No	Yes	Not simulated	-14.41
FOLR2	FOLR2_HUMAN	Yes	Yes	Not simulated	-18.45
FOLR1	FOLR1_HUMAN	Yes	Yes	-21.91	-20.41

FOLR3	FOLR3_HUMAN	No	Yes	Not simulated	-18.62
SLC19A1	S19A1_HUMAN	Yes	Yes	Not simulated	-13.38
MTHFS	MTHFS_HUMAN	Yes	Yes	Not simulated	-16.58
GNMT	GNMT_HUMAN	Yes	Yes	Not simulated	-14.05
NAALAD2	NALD2_HUMAN	Yes	Yes	-29.04	-14.97
NAALADL1	NALDL_HUMAN	Yes	No	-2.52	-9.55
NAALADL2	NADL2_HUMAN	No	No	Not simulated	-14.27

Note: Some proteins were not simulated due to the lack of confirmed 3D protein structures or due to the lack of expression on the plasma membrane such as SLC25A12, SLC25A13, SLC25A18 and SLC25A22 with expression on the mitochondrial membranes; or at neuro-synapses such as SLC1A2, SLC1A3, SLC1A6 and SLC1A7.
